# Angiotensin-converting enzyme inhibitors versus angiotensin II receptor blockers on insulin sensitivity in hypertensive patients: A meta-analysis of randomized controlled trials

**DOI:** 10.1371/journal.pone.0253492

**Published:** 2021-07-07

**Authors:** Jia Yao, Simin Fan, Xiaoyan Shi, Xiayu Gong, Jia Zhao, Guanjie Fan

**Affiliations:** 1 School of Second Clinical Medicine, Guangzhou University of Chinese Medicine, Guangzhou, China; 2 School of First Clinical Medicine, Guangzhou University of Chinese Medicine, Guangzhou, China; 3 School of Medicine, Southern University of Science and Technology, Shenzhen, China; 4 Research Center for Basic Integrative Medicine, Guangzhou University of Chinese Medicine, Guangzhou, China; 5 Department of Endocrinology, Guangdong Provincial Hospital of Chinese Medicine, The Second Affiliated Hospital of Guangzhou University of Chinese Medicine, Guangzhou, China; Shanghai Institute of Hypertension, CHINA

## Abstract

**Introduction:**

This meta-analysis aimed to summarize the available evidence to compare angiotensin-converting enzyme (ACE) inhibitors with angiotensin II receptor blockers (ARBs) on improving insulin sensitivity in hypertensive patients.

**Methods:**

Randomized controlled trials (RCTs) comparing ACE inhibitors versus ARBs published with outcomes on homeostasis model assessment of IR (HOMA-IR), glucose infusion rate (GIR), the quantitative insulin sensitivity check index (QUICKI), insulin sensitivity index (ISI) composite, fasting plasma glucose (FPG), fasting plasma insulin (FPI), systolic blood pressure (SBP), and diastolic blood pressure (DBP) were searched through 5 databases. Data were searched from their inception to July 5, 2020. Stata 14.0 was used to perform the meta-analysis.

**Results:**

Eleven RCTs (n = 1015) were included in this meta-analysis. Pooled analysis of studies showed no significant difference in HOMA-IR between ARBs and ACE inhibitors (WMD = -0.09, 95% CI: -0.69 to 0.50, P = 0.755); however, subgroup analysis of therapeutic duration showed a significant difference in HOMA-IR between ARBs and ACE inhibitors among the long-term intervention subgroup (>12 weeks) (WMD = 0.41, 95% CI: 0.06 to 0.76, P = 0.022) and hypertensive patients with diabetes mellitus subgroup (WMD = 0.55, 95% CI: 0.49 to 0.61, P < 0.001); results showed no significant difference between ARBs and ACE inhibitors on QUICKI score (WMD = -0.00, 95% CI: -0.03 to 0.03, P = 0.953) in hypertensive patients; however, the efficacy of ACE inhibitors on improving GIR and ISI composite was significantly better than that of ARBs (WMD = -1.09, 95% CI: -1.34 to -0.85, P < 0.001; WMD = -0.80, 95% CI: -1.24 to -0.36, P < 0.001, respectively). Furthermore, no significant differences were noted on FPG (WMD = 0.72, 95% CI: -1.39 to 2.83, P = 0.505), FPI (WMD = -0.48, 95% CI: -1.60 to 0.64, P = 0.398), SBP (WMD = -0.65, 95% CI: -1.76 to 0.46, P = 0.254), and DBP (WMD = -0.30, 95% CI: -1.70 to 1.10, P = 0.675) between ARBs and ACE inhibitors.

**Conclusion:**

Results from this meta-analysis showed that ACE inhibitors resulted in more effective improvement of HOMA-IR compared with ARBs among the long-term intervention and hypertensive patients with DM subgroup; furthermore, the efficacy of ACE inhibitors on improving GIR and ISI composite was significantly better than that of ARBs in hypertensive patients. However, ARBs had no significant difference in QUICKI score, FPG, FPI, SBP, and DBP compared with ACE inhibitors. Larger and better-designed studies are needed to further verify this conclusion.

## 1. Introduction

Insulin resistance (IR) can be defined as the inability of insulin to stimulate glucose disposal, and when IR occurs, insulin sensitivity (IS) will decrease, the sensitivity of tissues and target organs to insulin will decrease, and normal doses of insulin will not produce the normal hypoglycemic effect [1]. IR is considered as the common core pathological basis of metabolic disorders including hypertension, diabetes mellitus (DM), and metabolic syndrome (MS), which seriously threaten human health [2, 3]. Thus, improving IS represents one of the major pathways for drug development.

The close relationship between IR and the renin-angiotensin system (RAS) is not a recent observation. The overactivation of the RAS can lead to IR by affecting insulin signaling pathways, inhibiting fat formation, promoting oxidative stress and inflammation, reducing tissue blood flow, and activating the sympathetic nervous system [4–6]. Furthermore, the effect of RAS blockers including angiotensin-converting enzyme (ACE) inhibitors and angiotensin II (Ang II) receptor blockers (ARBs) on improving IS has gradually received attention. Experimental and clinical studies have shown that ACE inhibitors and ARBs can inhibit the activation of Ang II, improve blood perfusion, reduce oxidative stress and beta-cell apoptosis, and thus improve IS [3, 7]. However, although ACE inhibitors and ARBs both belong to the class of RAS blockers, differences between these two kinds of drugs exist. A meta-analysis compared studies that used ACE inhibitors or ARBs and found that the former was more effective in improving IS in hypertensive patients without diabetes [8]. However, this analysis was limited because only 4 randomized controlled trials (RCTs) with 203 hypertensive patients without DM were included. Therefore, more research is needed to better understand which class of drugs (ACE inhibitors or ARBs) has a stronger effect on IS in hypertensive patients.

To date, some RCTs have compared ACE inhibitors with ARBs on the efficacy of improving IS in hypertensive patients, however, the findings have been inconsistent. On this basis, we aim to conduct a meta-analysis of the available evidence to inform clinical practice.

## 2. Materials and methods

The current meta-analysis with its peer-reviewed protocol published online [9] was reported following the Preferred Reporting Items for Systematic Reviews and Meta-Analyses (PRISMA) statement. INPLASY registration number was INPLASY202050032.

### 2.1 Literature search

Four databases (PubMed, the Cochrane Library, Embase, and Web of Science) were searched for RCTs published from the database inception through July 5, 2020. Search terms were as follows: (“angiotensin-converting enzyme inhibitor” OR “ACE inhibitor” OR “ACEI”) AND (“angiotensin receptor antagonists” OR “angiotensin II type 1 receptor blockers” OR “angiotensin receptor blockers” OR ARB) AND (“hyperinsulinemic euglycemia clamp” OR “euglycemic clamp” OR “glucose clamp” OR HOMA OR “homeostasis model assessment” OR QUICKI OR “minimal model analysis” OR “minimal model” OR “index of insulin sensitivity” OR “insulin resistance” OR “insulin sensitivity”). The ClinicalTrials.gov registry was also searched for unpublished trials and the authors were contacted for additional information if necessary. Relevant references from included studies were sought to retrieve additional eligible studies. No limits were set on language, publication year, and type of publication.

### 2.2 Inclusion and exclusion criteria

The inclusion criteria were as follows: (1) RCTs published with any follow-up duration and sample size; (2) participants: hypertensive patients with or without other metabolic diseases (such as DM, IR, and MS); hypertension defined using the current and previously accepted definitions based on recommendations of the Joint National Committee on Prevention, Detection, Evaluation, and Treatment of High Blood Pressure [10–12]; we also adopted studies with a subjective definition of hypertension based on physician-diagnosed hypertension or use of antihypertensive medication due to elevated systolic or diastolic blood pressure measurement; DM and IR defined using the American Diabetes Association (ADA) or World Health Organization (WHO) criteria [13, 14]; MS defined using the current and previously accepted definitions [15–17]; age, gender, and other general conditions are not limited; (3) intervention: one group was given ARBs, the other group was given ACE inhibitors; and (4) studies that assessed IS using recognized methods such as the glucose clamp technique, homeostasis model assessment of IR (HOMA-IR), the quantitative insulin-sensitivity check index (QUICKI), or insulin sensitivity index (ISI) composite.

The exclusion criteria were as follows: (1) participants that did not meet the relevant diagnostic criteria; (2) interventions combined use of ACE inhibitors and ARB drugs, or treated with additional anti-hypertensive drugs, or studies involving other interventions; (3) outcome measures were not appropriate, relevant data could not be obtained from the original author; (4) non-randomized controlled trials, animal experiments or review articles; and (5) repeated published literature.

#### Outcomes

The primary outcome measure was IS. Several methods were used to assess IS, among them, the hyperinsulinemic-euglycemic clamp technique represents currently the ‘gold standard’ for quantifying IS in vivo because it directly measures the effects of insulin to promote glucose utilization under steady state conditions [18]; furthermore, alternatives for estimating IS include some simple surrogate indexes (e.g., QUICKI, HOMA-IR, ISI composite) that are derived from blood insulin and glucose concentrations under fasting conditions (steady state) or after an oral glucose load (dynamic) [19]. In our study, we included several methods (glucose clamp technique, HOMA-IR, QUICKI, and ISI composite) to estimate IS. The secondary outcomes were fasting plasma glucose (FPG), fasting plasma insulin (FPI), systolic blood pressure (SBP), and diastolic blood pressure (DBP).

### 2.3 Data extraction

Literature search and data extraction were performed by two researchers (J.Y. and S.F.) independently using predesigned forms, and the third researcher (X.S.) was involved in a discussion for any disagreements. The following information of eligible articles was extracted to a data extraction form: author, publication year, sample size, intervention, dosage, duration, mean age, body mass index (BMI), study population, and outcomes. When relevant details were insufficiently reported in studies, authors were contacted by email, and the ClinicalTrials.gov register was searched for further information.

### 2.4 Quality assessment

According to the Cochrane collaboration’s updated tool for assessing the risk of bias (version 5.1.0; updated March 2011), two reviewers (J.Y. and S.F.) assessed the quality of the included studies independently, and the senior reviewer (X.S.) was consulted for any disagreements. Each RCT was assigned a low, high, or unclear risk of bias for 6 specific domains (random sequence generation, allocation concealment, blinding of outcome assessment, blinding of participants and personnel, incomplete outcome data, selective outcome reporting, and other potential threats), using information identified from the published articles and supplementary materials and by contacting the study authors when needed.

### 2.5 Statistical analysis

Stata, version 14.0 (StataCorp LLC) was used for statistical analysis. To compare the effects of ARBs with ACE inhibitors on improving IS in patients; data for glucose infusion rate (GIR), QUICKI, HOMA-IR, ISI composite, FPG, FPI, SBP, and DBP were retrieved from the included RCTs. The mean and SD values of the ARBs group and ACEI inhibitors group were extracted to calculate the effect size. If SEs were reported rather than SDs, then SDs were calculated by equation $\mathrm{SD}=\mathrm{SE}\times \sqrt{n}$. If 95% CI was reported, SD was calculated by equation $\mathrm{SD}=\sqrt{n}$ ×(upper−lower)/2×t, where n is the number of subjects [20]. Continuous data (HOMA-IR, GIR, QUICKI, ISI composite, FPG, FPI, SBP, and DBP) used the weighted mean difference (WMD) with 95% CI after the units were standardized [21]. Heterogeneity was tested by χ^2^-based Cochran Q statistic (P < 0.10 indicated statistically significant heterogeneity) and I^2^ statistic. If I^2^ < 50%, a fixed-effects model was used to pool the estimations across studies. If I^2^ ≥ 50%, after excluding clinical heterogeneity between studies, the random-effects model was used. Subgroup and sensitivity analyses were conducted to explore potential sources of heterogeneity, to assess the reliability and stability of the pooled results. Sensitivity analysis was performed by excluding low-quality studies, trials recruiting participants with particular conditions or trials with characteristics different from the others. When possible and appropriate, planned subgroup analyses included the therapeutic duration, sample size, and study population of the included studies. The funnel plot and Egger’s and Begg’s tests were used to judge publication bias, and the trim and fill method was used to correct the funnel asymmetry caused by publication bias. P < 0.05 was considered statistically significant.

## 3. Results

### 3.1 Search results

**Fig 1:**
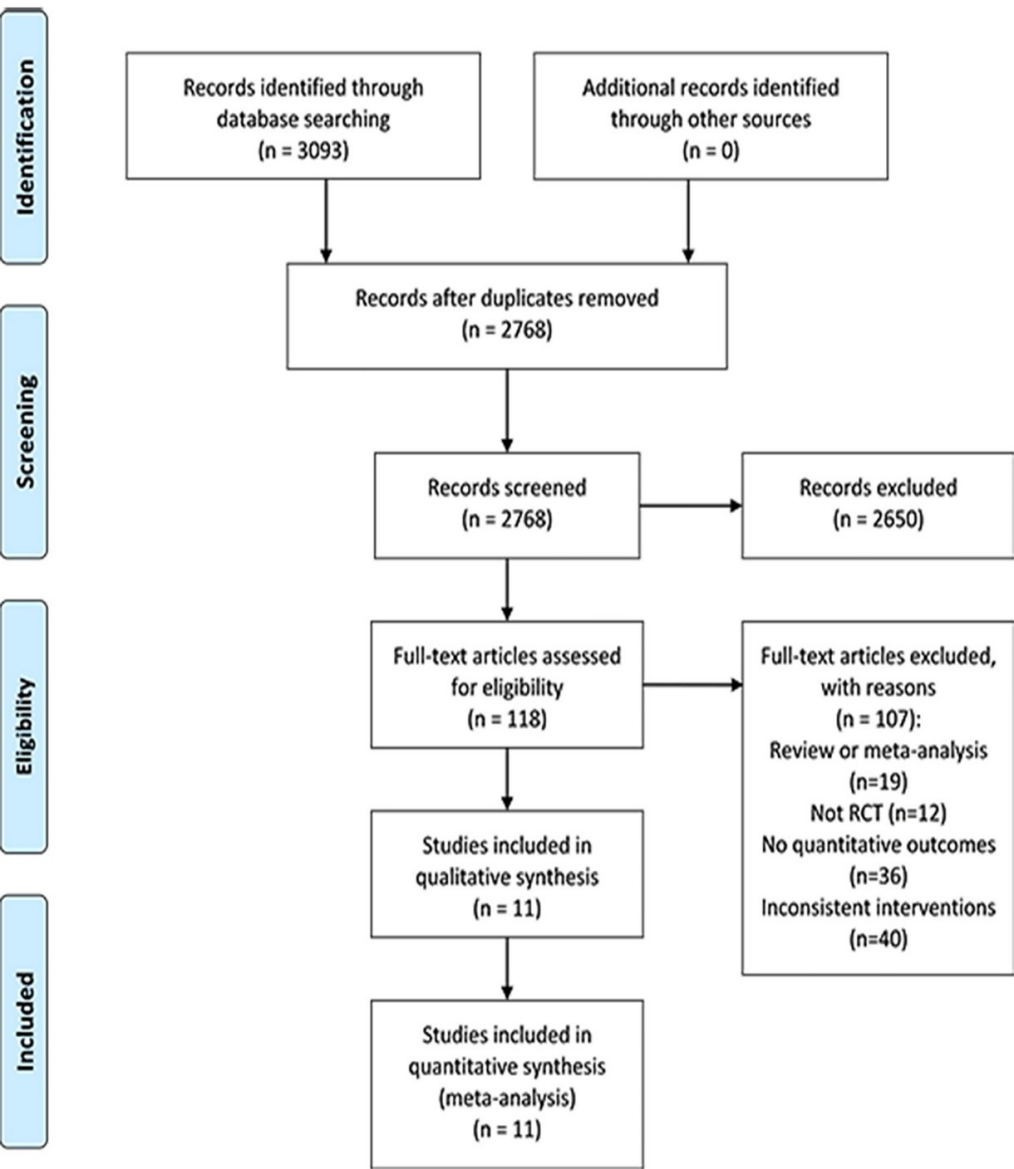
Flow diagram of study selection.

As displayed in Fig 1, in total, we identified 3093 citations with 325 duplicates. After preliminary screening of the titles and abstracts, 118 studies were selected for full-text review, and then 107 studies were excluded since 19 of them were reviews or meta-analyses, 12 of them were not RCTs, 36 studies didn’t provide quantitative outcomes, and the rest were those with undesirable interventions. Correspondence with the authors via e-mail was done to obtain the needed information for the study with no specific data on an outcome. Unfortunately, no reply from the authors was obtained until the time of this writing. Ultimately, 11 RCTs [22–32] were determined to be included in this meta-analysis.

### 3.2 Study characteristics

Eleven studies involving 1015 subjects were included in this meta-analysis. Sample size ranged from 18 to 466 participants, duration varied from 6 weeks to 12 months, mean age ranged from 33.0 to 59.7 years, BMI varied from 23.8 to 33.4 kg/m^2^, 1 (9%) RCT included hypertensive patients with left ventricular hypertrophy, 1 (9%) RCT included hypertensive patients with IR, 1 (9%) RCT included hypertensive patients with DM, 1 (9%) RCT included hypertensive postmenopausal women, 6 (55%) RCTs included patients with hypertension, 1 (9%) RCT included hypertensive patients with MS. A total of 7 (64%) RCTs reported HOMA-IR as an outcome, 2 (18%) RCTs reported QUICKI as an outcome, 2 (18%) RCTs reported GIR as an outcome, 1 (9%) RCT reported ISI composite as an outcome (Table 1). Changes of outcome measures extracted from excluded studies are summarized in Table 2.

**Table 1:** Baseline characteristics of trials included in the analysis.

Author, year	Groups	Sample size	Dosage (mg qd)	Durat-ion	Mean age (year)	BMI (kg/m^2^)	Study Population	IS measure
Anan (2005) [22]	Valsartan	10	160	40w	59.0±8.0	25.5±1.0	Hypertension with LVH	HOMA-IR
	Perindopril	11	8		59.0±7.0	25.4±1.0		
Brown (2002) [23]	Losartan	11	100	6w	47.9±8.3	31.2±1.7	Hypertension with IR	HOMA-IR
	Ramipril	9	10		47.8±10.8	33.4±4.2		
Derosa (2003) [24]	Candesartan	47	16	12m	55±9.0	26.8±2.5	Hypertension with DM	HOMA-IR
	Perindopril	49	4		53±10.0	27.2±2.0		
Fogari (2001) [25]	Losartan	44	50	12w	56.1±2.0	25.9±2.1	Hypertensive postmenopausal women	GIR
	Trandolapril	45	2		55.7±2.0	26.1±2.2		
Fogari (2011) [26]	Candesartan	28	8	12w	53.2±9.7	23.9±1.1	Hypertension	GIR
	Imidapril	28	5		53.0±10.8	23.8±1.1		
Gilowski (2018) [27]	Telmisartan	26	40	6w	49±12.0	28.1±4.3	Hypertension	HOMA-IR
	Perindopril	26	4		45±10.0	27.8±3.9		
Koh (2007) [28]	Candesartan	34	16	2m	46±11.7	25.2±2.5	Hypertension	QUICKI
	Ramipril	34	10		46±11.7	25.2±2.5		
Koh (2010) [29]	Candesartan	31	16	8w	47±11.1	25.2±2.4	Hypertension	QUICKI
	Ramipril	30	10		46±5.5	25.1±2.5		
Napoli (2016) [30]	Irbesartan	235	300	24w	59.7±9.3	31.3±4.8	Hypertension with MS	HOMA-IR
	Zofenopril	231	60		58.5±9.8	31.3±4.7		
Sanchez (2008) [31]	Telmisartan	34	80	3m	33.0±4.5	28.5±4.0	Hypertension	HOMA-IR
	Ramipril	34	10		33.0±4.5	28.5±4.0		
Yavuz (2003) [32]	Losartan	9	50 to 100	6m	42.2±12.8	24.4±4.5	Hypertension	ISI composite; HOMA-IR
	Enalapril	9	5 to 40		38.6±7.9	24.7±4.9		

BMI, body mass index; IS, insulin sensitivity; M, month; w, week; LVH, left ventricular hypertrophy; IR, insulin resistance; DM, diabetes mellitus; MS, metabolic syndrome; HOMA-IR, homeostasis model assessment of insulin resistance; GIR, glucose infusion rate; QUICKI, quantitative insulin sensitivity check index; ISI, insulin sensitivity index.

**Table 2:** Changes of outcomes in individual studies.

Author, year	Groups		IS			FPG		FPI		SBP		DBP	
				B	A	B	A	B	A	B	A	B	A
Anan (2005) [22]	Valsartan		HOMA-IR	2.4±0.6	2.1±0.6	6.8±0.9	6.4±0.9	8.0±1.0	7.1±1.2	157±7	134±7	97±7	85±7
	Perindopril			2.3±0.6	1.9±0.6	6.6±0.8	6.1±0.9	7.9±0.9	6.9±1.0	156±9	133±6	97±6	85±5
Brown (2002) [23]	Losartan			3.6±0.7*	4.2±0.8*	5.6±0.2*	5.8±0.2*	101±21*	119±24*	140.6±4.9*	123.7±2.6*	96.9±2.2*	86.4±2.1*
	Ramipril			3.4±0.8*	4.8±1.1*	5.5±0.4*	5.4±0.2*	166±68*	137±28*	144.8±3.8*	127.0±3.1*	98.6±2.5*	91.4±3.3*
Derosa (2003) [24]	Candesartan			3.99±2.5	-0.25±0.08	160±13	-8±2	10.6±6.1	-0.7±0.4	148±6	-12±4.1	93±5	-8±2.9
	Perindopril			3.86±2.2	-0.8±0.2	155±15	-15±4	10.2±5.8	-1.4±0.9	147±6	-13±4.5	94±4	-11±3.6
Gilowski (2018) [27]	Telmisartan			3.1±1.9	2.6±1.6	101±15	97±12	/	/	154±15	136±13	93±7	85±8
	Perindopril			2.6±1.1	2.4±1.2	97±7	95±8	/	/	149±12	136±14	90±8	84±7
Napoli (2016) [30]	Irbesartan			3.7±3.7	0.2 (-0.6,1.1) ^**#**^	117.4±40	2.6 (-3.4,8.7) ^**#**^	13.8±11.4	0.2 (-2.0,2.3) ^**#**^	132.9±14.2	-18.8 (21.0,16.6) ^**#**^	83.4±8.5	-10.4 (11.8,9.0) ^**#**^
	Zofenopril			4.1±5.3	0.5 (-0.3,1.3) ^**#**^	117.9±42	1.9 (-4.1,7.9) ^**#**^	14.6±15.6	1.1 (-1.1,3.2) ^**#**^	131.7±14.6	-17.0 (19.2,14.8) ^**#**^	83.9±10.0	-9.8 (11.1,8.4) ^**#**^
Sanchez (2008) [31]	Telmis-artan	MHT		2.76±0.16	2.24±0.18	92±8	94±2.8	9.2±2	8.8±1.3	154±8	137±6	96±5	88±4
		NMHT		4.4±1	2.3±0.7	99±10	88±8.8	16±4	8.4±2	161±9	137±5	96±5	86±3
	Ramipril	MHT		2.76±0.16	2.6±0.75	92±8	89±8	9.2±2	9.0±2	159±10	142±6	102±4	93±3
		NMHT		4.4±1	4.2±0.7	99±10	96±5.2	16±4	14±5.6	162±12	139±7	97±4	89±2
Yavuz (2003) [32]	Losartan			2.3±0.6	1.5±0.7	/	/	/	/	150±21	126±14	100±5	80±2
	Enalapril			2.9±1.7	1.2±0.6	/	/	/	/	149±11	126±11	98±7	79±3
Fogari (2001) [25]	Losartan		GIR	6.74±0.47	6.96±0.50	93±9	92±10	77±39	/	160.6±12	145.4±11	100.5±5	88.6±5
	Trandolapril			6.67±0.56	7.99±0.65	92±10	89±10	74±36	/	162.1±12	145.2±10	101.2±5	88.1±4
Fogari (2011) [26]	Candesartan			5.2±1.8	5.3±1.7	89.1±8.9	89.2±8.8	9.5±2.8	9.4±2.7	149.0±4.9	132.9±4.4	98.5±4.0	86.3±3.2
	Imidapril			5.2±2.0	6.3±1.8	88.9±8.8	88.4±8.7	9.4±2.7	9.1±2.6	148.4±4.8	132.4±4.1	98.7±4.4	86.1±2.6
Koh (2007) [28]	Candesartan		QUICKI	0.406±0.011*	0.423±0.011*	85±2*	84±3*	4.68±0.42*	4.28±0.57*	156±1*	137±2*	95±1*	85±1*
	Ramipril			0.428±0.023*	0.448±0.026*	84±2*	85±2*	4.38±0.51*	4.02±0.53*	155±1*	142±2*	95±1*	88±2*
Koh (2010) [29]	Candesartan			0.348±0.008*	0.362±0.008*	104±2*	101±3*	9.70±0.88*	8.42±1.10*	156±1*	136±2*	94±1*	84±1*
	Ramipril			0.382±0.016*	0.396±0.018*	101±2*	103±3*	8.42±1.04*	7.55±1.05*	155±1*	143±2*	94±1*	87±1*
Yavuz (2003) [32]	Losartan		ISI composite	1.1±0.3	1.3±0.4	/	/	/	/	150±21	126±14	100±5	80±2
	Enalapril			0.9±0.3	1.9±0.6	/	/	/	/	149±11	126±11	98±7	79±3

Data are shown as mean ± SD

* Data are shown as mean ± SE

^#^ Data are shown as mean changes (95% confidence interval).

Abbreviations: IS, insulin sensitivity; FPG, fasting plasma glucose; FPI, fasting plasma insulin; SBP, systolic blood pressure; DBP, diastolic blood pressure; B, before treatment; A, after treatment; MHT: modulating hypertension; NMHT: nonmodulating hypertension; HOMA-IR, homeostasis model assessment of insulin resistance; GIR, glucose infusion rate; QUICKI, quantitative insulin sensitivity check index; ISI, insulin sensitivity index.

### 3.3 Quality assessment

The risk of bias data for the included RCTs is presented in Table 3. Randomization was categorized as low risk in 1 (9%) RCT with appropriate use of random sequence generation. The remaining 10 (91%) studies did not provide details about the method of randomization and were categorized as unclear risk. Allocation concealment was categorized as low risk in 2 (18%) studies with a detailed description. The remaining 9 (82%) studies were categorized as unclear risk due to no relevant description. Furthermore, 5 (45%) studies were conducted using the double-blinded method, 1 (9%) study followed in an unblinded fashion, 1 (9%) study was conducted using the open-label, blinded-endpoint method, and the remaining studies did not mention the blinding of participants, personnel, and outcome assessment. Incomplete outcome data were categorized as low risk in 11 (100%) studies with no missing outcome data or reasons for missing outcome data balanced in numbers across intervention groups. As for selective reporting, 7 (64%) studies with all expected outcomes, the remaining 4 (36%) were classified as an unclear risk because of insufficient information to permit judgment of “low risk” or “high risk”. As for other bias, 11 (100%) studies were classed as low risk.

**Table 3:** Risk of bias assessment in the included studies.

Study (year)	Random sequence generation	Allocation concealment	Blinding of participants and personnel	Blinding of outcome assessment	Incomplete outcome data	Selective reporting	Other bias
Anan (2005) [22]	U	U	U	U	L	L	L
Brown (2002) [23]	U	U	U	U	L	U	L
Derosa (2003) [24]	U	L	L	L	L	L	L
Fogari (2001) [25]	U	U	L	L	L	L	L
Fogari (2011) [26]	U	U	H	L	L	L	L
Gilowski (2018) [27]	U	U	U	U	L	U	L
Koh (2007) [28]	U	U	L	L	L	L	L
Koh (2010) [29]	U	L	L	L	L	L	L
Napoli (2016) [30]	U	U	L	L	L	L	L
Sanchez (2008) [31]	U	U	U	U	L	U	L
Yavuz (2003) [32]	L	U	H	H	L	U	L

H, high risk; L, low risk; U, unclear risk.

### 3.4 Pooled results

#### 3.4.1 ARBs versus ACE inhibitors on IS

**Fig 2:**
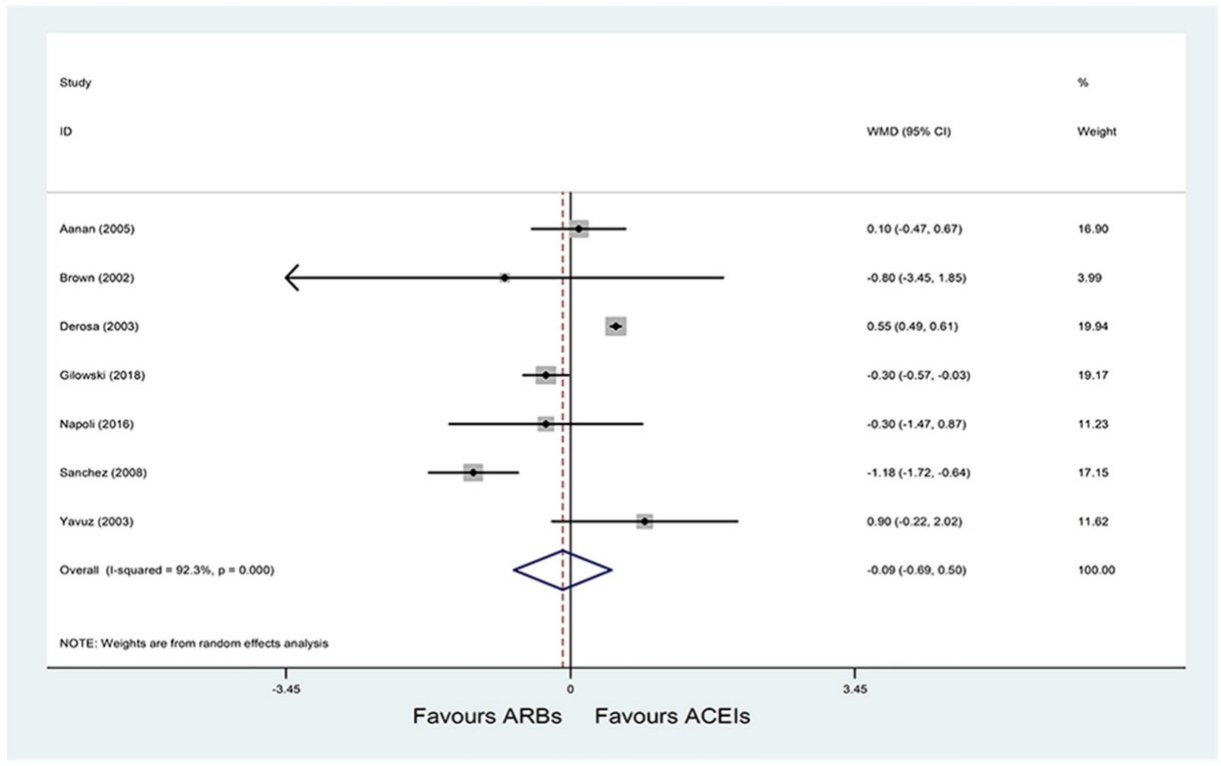
Effect of ARBs versus ACE inhibitors on HOMA-IR.

*(1) HOMA-IR*. A total of 7 RCTs [22–24, 27, 30–32] with 664 patients reported HOMA-IR as an outcome, and significant heterogeneity was observed (P < 0.001; I^2^ = 92.3%). Pooled results with a random-effects model showed that ARBs had no significant difference on HOMA-IR compared with ACE inhibitors (WMD = -0.09, 95% CI: -0.69 to 0.50, P = 0.755) (Fig 2). Results of sensitivity and subgroup analysis were shown in Table 4. Sensitivity analysis showed that after excluding Derosa et al. [24] and Sanchez et al. [31], heterogeneity was decreased (P = 0.240; I^2^ = 27.2%). Pooled results with a fixed-effects model showed that the HOMA-IR still didn’t differ in two groups (WMD = -0.18, 95% CI: -0.42 to 0.05, P = 0.123). Subgroup analysis was performed based on the therapeutic duration (< = 12 weeks or > 12 weeks), sample size (< = 80 or > 80), and study population (hypertension with left ventricular hypertrophy, hypertension with IR, hypertension with DM, hypertension, or hypertension with MS). The subgroup analysis did not show any significant differences within subgroups based on sample size. However, subgroup analysis of therapeutic duration showed a significant difference in HOMA-IR between ARBs and ACE inhibitors group among the long-term intervention subgroup (> 12 weeks) (WMD = 0.41, 95% CI: 0.06 to 0.76, P = 0.022) rather than the short-term intervention subgroup (< = 12 weeks) (WMD = -0.71, 95% CI: -1.47 to 0.05, P = 0.069). Furthermore, results showed a significant difference in HOMA-IR between two groups in hypertensive patients with DM (WMD = 0.55, 95% CI: 0.49 to 0.61, P < 0.001), but there was no significant difference between the two groups among hypertensive patients, hypertensive patients with left ventricular hypertrophy, hypertensive patients with IR, and hypertensive patients with MS (Table 4).

**Table 4:** Sensitivity analysis and subgroup analysis.

	No. of trial	WMD	95% CI	P	I^2^; P	Effect model
**Sensitivity analysis**						
Derosa (2003) [24] and Sanchez (2008) [31] removed	5	-0.18	-0.42 to 0.05	0.123	27.2%; 0.240	FE
**Subgroup analysis**						
Therapeutic duration						
Duration < = 12 w	3	-0.71	-1.47 to 0.05	0.069	75.7%;0.016	RE
Duration > 12 w	4	0.41	0.06 to 0.76	0.022	37.5%; 0.187	RE
Sample size						
Sample size < = 80	5	-0.27	-0.86 to 0.33	0.384	75.9%; 0.002	RE
Sample size > 80	2	0.33	-0.39 to 1.06	0.369	50.8%; 0.154	RE
Study population						
Hypertension with LVH	1	0.10	-0.47 to 0.67	0.729	/	RE
Hypertension with IR	1	-0.80	-3.45 to 1.85	0.554	/	RE
Hypertension with DM	1	0.55	0.49 to 0.61	<0.001	/	RE
Hypertension	3	-0.32	-1.17 to 0.53	0.454	85.4%; 0.001	RE
Hypertension with MS	1	-0.30	-1.47 to 0.87	0.614	/	RE

w, weeks; LVH, left ventricular hypertrophy; IR, insulin resistance; DM, diabetes mellitus; MS, metabolic syndrome; FE, fixed-effects model; RE, random-effects model.

**Fig 3:**
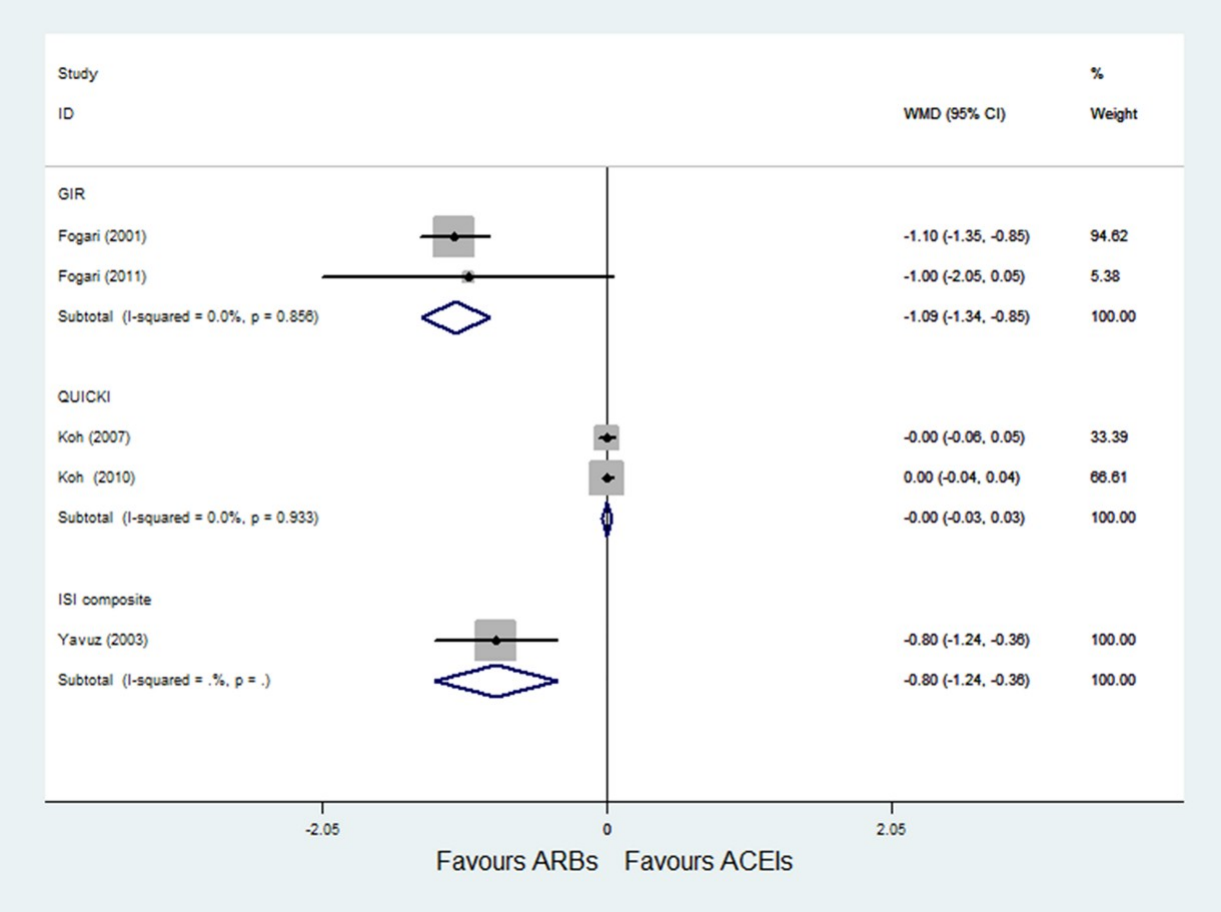
Effect of ARBs versus ACE inhibitors on GIR, QUICKI, and ISI composite.

*(2) GIR*. A total of 2 RCTs [25, 26] with 145 patients reported GIR as an outcome, and no heterogeneity was observed (P = 0.856; I^2^ = 0.0%). Pooled results with a fixed-effects model showed that the efficacy of ACE inhibitors on improving GIR was significantly better than that of ARBs (WMD = -1.09, 95% CI: -1.34 to -0.85, P < 0.001) (Fig 3).

*(3) QUICKI*. A total of 2 RCTs [28, 29] with 129 patients reported QUICKI as an outcome, and no heterogeneity was observed (P = 0.933; I^2^ = 0.0%). Pooled results with a fixed-effects model showed that ARBs had no significant difference on QUICKI compared with ACE inhibitors (WMD = -0.00, 95% CI: -0.03 to 0.03, P = 0.953) (Fig 3).

*(4) ISI composite*. A total of 1 RCTs [32] with 18 patients reported ISI composite as an outcome. Pooled results with a fixed-effects model showed that the efficacy of ACE inhibitors on improving ISI composite was significantly better than that of ARBs (WMD = -0.80, 95% CI: -1.24 to -0.36, P < 0.001) (Fig 3).

#### 3.4.2 ARBs versus ACE inhibitors on FPG

**Fig 4:**
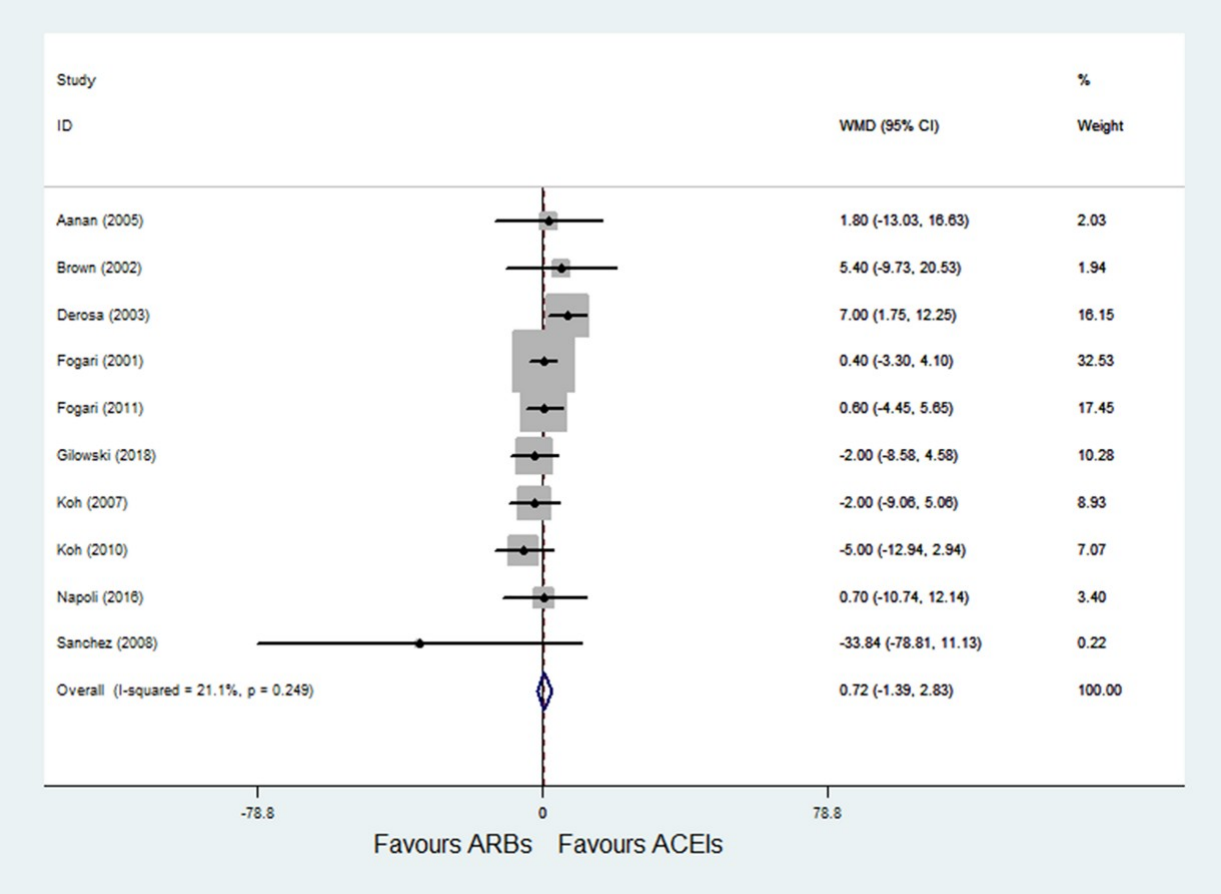
Effect of ARBs versus ACE inhibitors on fasting plasma glucose.

A total of 10 RCTs [22–31] with 955 patients reported FPG as an outcome, and small heterogeneity was observed (P = 0.249; I^2^ = 21.1%). Pooled results with a fixed-effects model showed that ARBs had no significant difference on FPG compared with ACE inhibitors (WMD = 0.72, 95% CI: -1.39 to 2.83, P = 0.505) (Fig 4).

#### 3.4.3 ARBs versus ACE inhibitors on FPI

**Fig 5:**
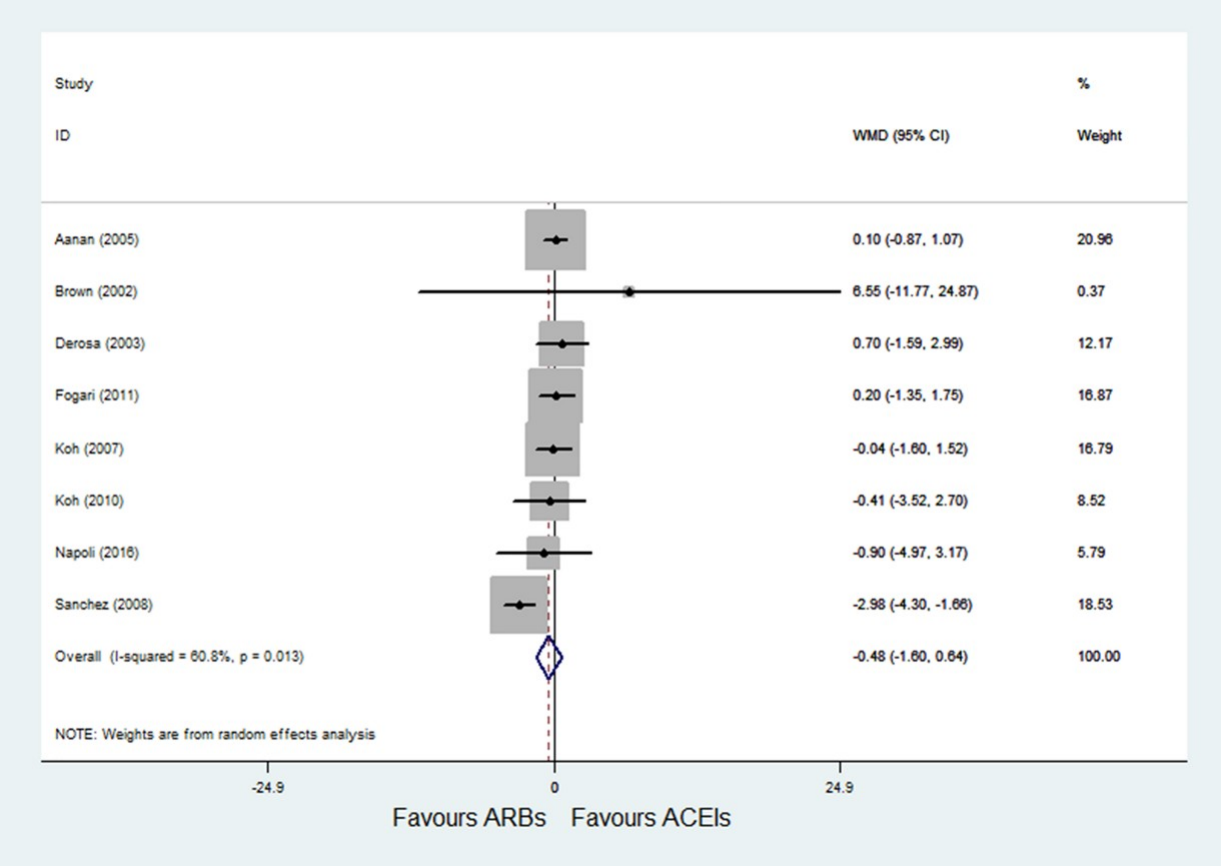
Effect of ARBs versus ACE inhibitors on fasting plasma insulin.

A total of 8 RCTs [22–24, 26, 28–31] with 787 patients reported FPI as an outcome, and significant heterogeneity was observed (P = 0.013; I^2^ = 60.8%). Pooled results with a random-effects model showed that ARBs had no significant difference on FPI compared with ACE inhibitors (WMD = -0.48, 95% CI: -1.60 to 0.64, P = 0.398) (Fig 5). After excluding Sanchez et al. [31], heterogeneity was decreased (P = 0.981; I^2^ = 0.0%); and the results of sensitivity analysis were not altered after excluding the trial by Sanchez et al. [31] (WMD = 0.10, 95% CI: -0.57 to 0.77, P = 0.765).

#### 3.4.4 ARBs versus ACE inhibitors on SBP

**Fig 6:**
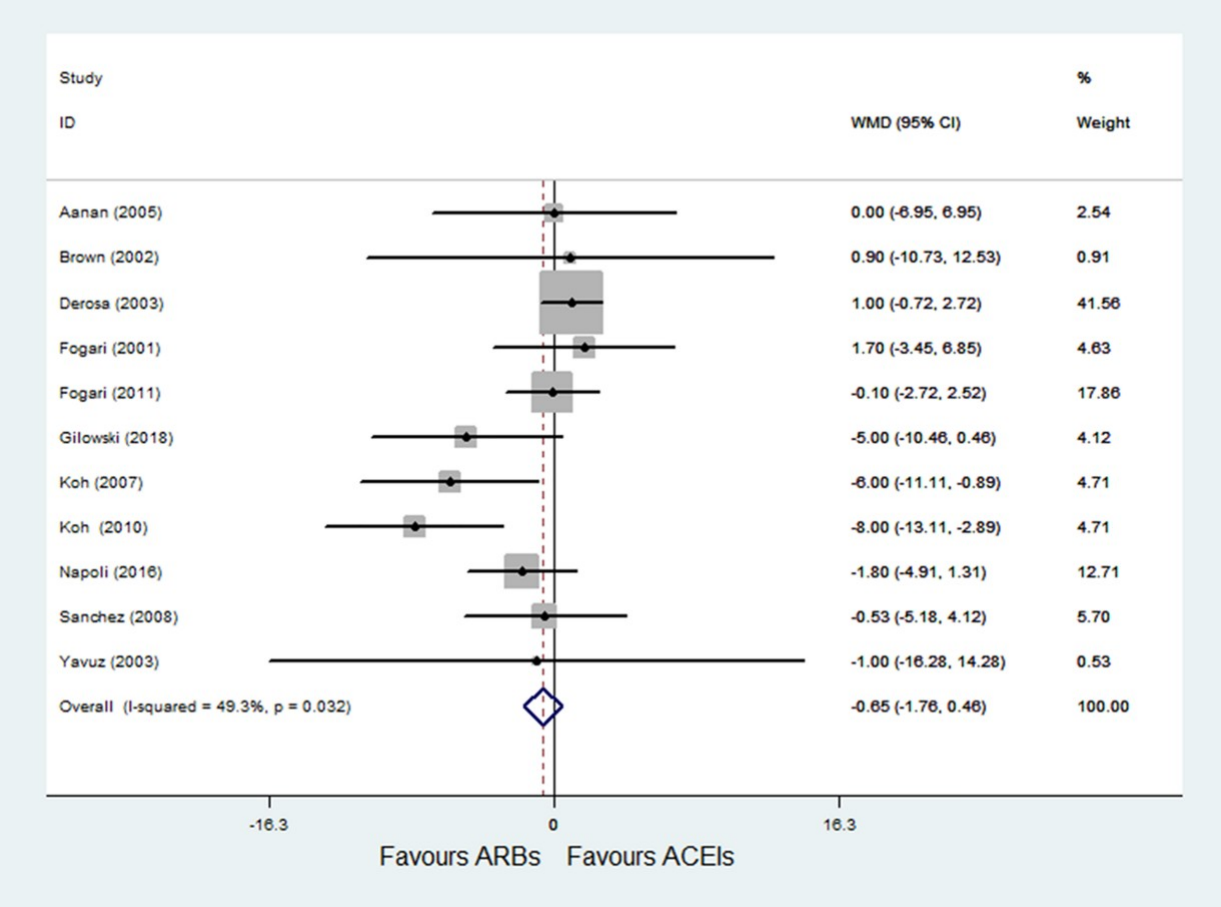
Effect of ARBs versus ACE inhibitors on systolic blood pressure.

A total of 11 RCTs [22–32] with 1015 patients reported SBP as an outcome, and moderate heterogeneity was observed (P = 0.032; I^2^ = 49.3%). Pooled results with a fixed-effects model showed that ARBs had no significant difference compared with ACE inhibitors on SBP (WMD = -0.65, 95% CI: -1.76 to 0.46, P = 0.254) (Fig 6).

#### 3.4.5 ARBs versus ACE inhibitors on DBP

**Fig 7:**
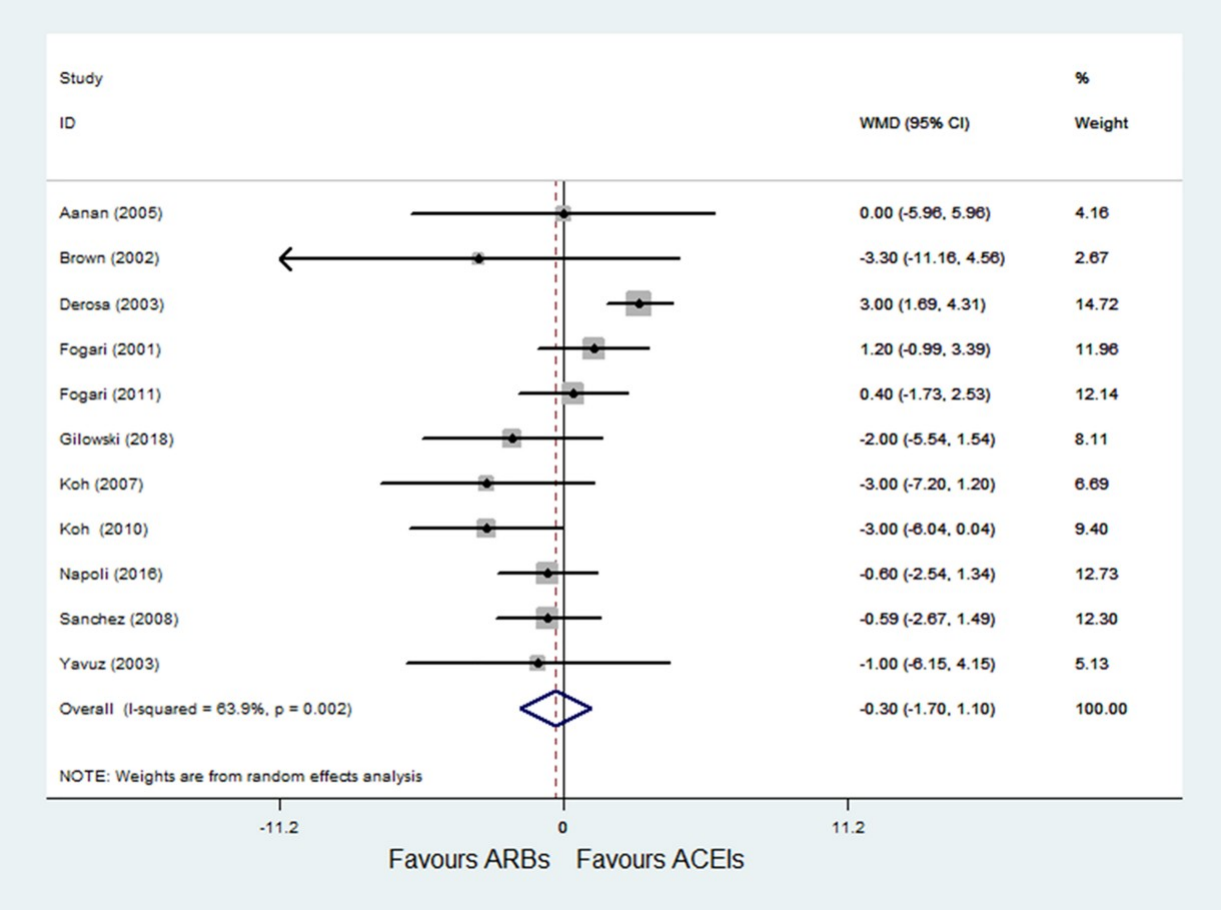
Effect of ARBs versus ACE inhibitors on diastolic blood pressure.

A total of 11 RCTs [22–32] with 1015 patients reported DBP as an outcome, and obvious heterogeneity was observed (P = 0.002; I^2^ = 63.9%). Pooled results with a random-effects model showed that ARBs had no significant difference compared with ACE inhibitors on DBP (WMD = -0.30, 95% CI: -1.70 to 1.10, P = 0.675) (Fig 7). After excluding Derosa et al. [24], heterogeneity was decreased (P = 0.513; I^2^ = 0.0%); and the results of sensitivity analysis were not altered after excluding the trial by Derosa et al. [24] (WMD = -0.56, 95% CI: -1.45 to 0.33, P = 0.220).

#### 3.4.6 Publication bias

**Fig 8:**
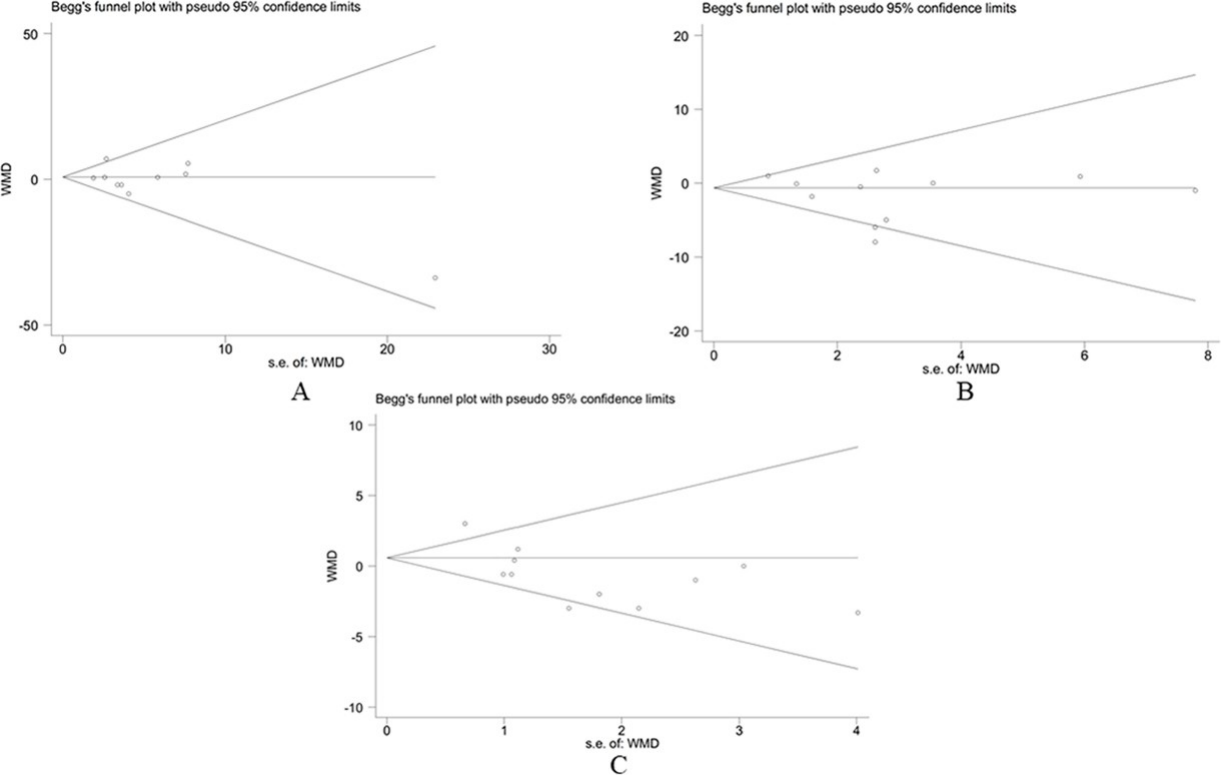
Publication bias analysis of fasting plasma glucose, systolic blood pressure, and diastolic blood pressure. (A) fasting plasma glucose, (B) systolic blood pressure, (C) and diastolic blood pressure.

Publication bias analysis was conducted on the outcome of FPG, SBP, and DBP. The funnel plots were symmetrical, most scatter points were inside the confidence limit, and the p-value of Begg’s tests were 1.000, 0.640, and 0.640, respectively. As shown in Fig 8, each meta-analysis did not show significant publication bias.

## 4. Discussion

The present study focused on the effects of ARBs versus ACE inhibitors on IS, FPG, FPI, SBP, and DBP in hypertensive patients for resolving the conflicting results of the outcomes of earlier studies. In a meta-analysis done by Yang et al. [8], where the improvement of IS was compared among patients on ACE inhibitors versus ARBs, ACE inhibitors were shown to have a significant effect on improving IS in hypertensive patients without diabetes. However, this analysis was limited because it only included 4 RCTs with 203 hypertensive patients without diabetes, other study populations such as hypertensive patients with DM, IR, or MS were not included; HOMA-IR outcome in the patients was not measured, and the results of the more recent RCTs were not yet included in the analysis. Therefore, the main findings of our study and this study were different from each other.

Eleven studies [22–32] involving 1015 subjects were finally included in the present meta-analysis. To examine the IS, those studies that investigated HOMA-IR, GIR, QUICKI index, and ISI composite were entered into the meta-analysis. Pooled results showed that ARBs had no significant difference on HOMA-IR compared with ACE inhibitors in general (95% CI: -0.69 to 0.50, P = 0.755). However, heterogeneity of these studies in this area was high (P < 0.001; I^2^ = 92.3%). With the heterogeneity, we noted that the eligible trials varied in several respects, including differences in the study population, baseline comorbidities, intervention drugs, and methodological differences, which may contribute to substantial heterogeneity. Sensitivity analysis showed that after excluding Derosa et al. [24] and Sanchez et al. [31], heterogeneity was decreased (P = 0.240; I^2^ = 27.2%), pooled results with a fixed-effects model showed that the HOMA-IR still didn’t differ in two groups. The study with long-term intervention (12 months) conducted by Derosa et al. [24] included patients with hypertension and DM, and the RCT conducted by Sanchez et al. [31] was a crossover study and included both modulating and non-modulating hypertensive patients; these were how they differ from other studies and may be the cause of heterogeneity. Subgroup analysis of therapeutic duration showed that ACE inhibitors resulted in more effective improvement of HOMA-IR compared with ARBs among the long-term intervention subgroup (> 12 weeks) (P = 0.022) rather than the short-term intervention subgroup (< = 12 weeks) (P = 0.069). Furthermore, Subgroup analysis of the study population showed that ACE inhibitors showed an improvement on HOMA-IR compared with ARBs in hypertensive patients with DM (P < 0.001). For other IS indicators, pooled results showed no significant difference between ARBs and ACE inhibitors on QUICKI score in hypertensive patients; however, the efficacy of ACE inhibitors on improving GIR and ISI composite was significantly better than that of ARBs in hypertensive patients or hypertensive postmenopausal women.

In the meta-analysis of FPG and FPI, results showed that ARBs had no significant difference on FPG and FPI compared with ACE inhibitors. However, significant heterogeneity was observed in FPI outcome (P = 0.013; I^2^ = 60.8%); with the heterogeneity, we noted that Sanchez et al. [31] was a crossover study and included both modulating and non-modulating hypertensive patients. After excluding Sanchez et al. [28], the heterogeneity was eliminated (P = 0.981; I^2^ = 0.0%), and pooled results with a fixed-effects model showed that the FPI still didn’t differ in the two groups.

In the meta-analysis of SBP and DBP, results showed that ARBs had no significant difference on SBP and DBP compared with ACE inhibitors. However, significant heterogeneity was observed in DBP outcome (P = 0.002; I^2^ = 63.9%); after excluding the study conducted by Derosa et al. [24] which had a long-term intervention (12 months) and included patients with hypertension and DM, the heterogeneity was eliminated (P = 0.513; I^2^ = 0.0%), and pooled results with a fixed-effects model showed that the DBP outcome still didn’t differ in two groups.

Studies have revealed that the RAS is closely related to IR, and the overactivation of the RAS can lead to IR in the following ways: (1) Ang II can promote IR by affecting insulin signaling pathways, inhibiting fat formation, promoting oxidative stress and inflammation, reducing tissue blood flow, and activating sympathetic nervous system [4–6]. (2) Hyperinsulinemia associated with IR can activate RAS, increase the expression of angiotensinogen, Ang II, and AT1 receptors, and further aggravate IR [33]. (3) Another mechanism is through aldosterone, which is also a regulator of salt and water balance and is involved in IR by inhibiting the expression of insulin receptor and glucose transporters, as well as by degrading insulin receptor substrate or inhibiting the insulin signaling pathway [34]. (4) Furthermore, the ACE2/ANG (1–7)/MAS receptor axis functions as a negative regulator of the classical RAS, which was recently discovered and is responsible for improving IS by antagonizing the biological effect of Ang II. Ang (1–7) can also improve IS via reducing oxidative stress through MAS receptor activation possibly via improved adiponectin secretion [35]. Furthermore, IR and RAS activation aggravate each other and a vicious IR-RAS activation-inflammation/endothelial dysfunction-IR cycle is present in patients with various metabolic disorders. Thus, breaking this vicious circle is crucial to the prevention and treatment of metabolic diseases.

The effects of RAS blockers on improving IS have gradually received attention, and massive studies have shown that RAS blockers could improve IS [7]. ACE inhibitors and ARBs are two kinds of RAS system blockers and were initially used as antihypertensive drugs in the clinic. ACE inhibitors and ARBs are both equally effective in lowering blood pressure and have beneficial effects on glucose metabolism. Therefore, the clinical guidelines recommend these two types of drugs for the first-line treatment of hypertension with diabetes [36]. Although substantial studies have confirmed the beneficial effects of ACE inhibitors and ARBs on the improvement of IS [3, 7], differences between the two kinds of RAS system blockers exist. Results from the present meta-analysis showed that ARBs had no significant difference on QUICKI score, FPG, FPI, SBP, and DBP compared with ACE inhibitors; however, ACE inhibitors resulted in more effective improvement of HOMA-IR compared with ARBs among the long-term intervention subgroup and hypertensive patients with DM subgroup; furthermore, the efficacy of ACE inhibitors on improving GIR and ISI composite was significantly better than that of ARBs in hypertensive patients or hypertensive postmenopausal women. Studies have shown that ARBs can bind to AT1 receptors with high affinity selectively and competitively, thus inhibiting the activation of Ang II, improving blood perfusion, reducing oxidative stress and beta-cell apoptosis, and accordingly improving IR [7]. In addition to inhibiting the conversion of angiotensinogen I into Ang II which can reduce the generation of Ang II and improves IS [3, 7], ACE inhibitors can improve IS in the following additional ways: (1) ACE inhibitors may be mediated by the increased bradykinin levels by inhibiting kallikrein II, which not only could enhance insulin signaling and translocation of the glucose transporter, GLUT-4 in skeletal muscle, but also could directly increase nitric oxide levels, which enhance insulin-stimulated glucose oxidation and transport [37]. Shiuchi et al [38] also verified ACE inhibitors improved IS by enhancing bradykinin and nitric oxide in the diabetic mouse. Indeed, some studies suggest that the positive effect of ACE inhibition on IR is predominantly mediated by increased bradykinin actions via the B2kinin receptor and therefore may not be observed with AT1 receptor blockade [39]. (2) ACE inhibitors could increase adiponectin and leptin concentrations and decrease TNF- α levels, substances that are believed to enhance IS [40]. (3) ACE inhibitors could improve IS by relaxing smooth muscle, promoting water and sodium excretion, and reducing sympathetic nervous system excitability [41]. (4) In addition, the favored ACE2 axis may mediate these additional effects of improving IS from ACE inhibitors through enhanced ANG (1–7) action [42]. The beneficial effects of ACE inhibitors rely on a higher ACE2/ACE ratio and reduce oxidative stress and glucotoxicity by improving glucose-stimulated insulin secretion and islet function while reducing oxidative stress and inflammation [7]. To sum up, we hypothesize that the additional ways mentioned above may represent a major reason why ACE inhibitors demonstrated a stronger effect of improving HOMA-IR, GIR, and ISI composite than ARBs in the current study. Furthermore, the absence of significant differences between ACE inhibitors and ARBs on QUICKI in this study does not mean that there is no evidence of a true difference. Only 2 RCTs [28, 29] were included, the relatively limited data for ACE inhibitors in comparison with ARBs and the short-term intervention (8 weeks) could still be underpowered to detect a true difference.

There are also limitations of the current analysis that should be taken into consideration. Firstly, the number of RCTs currently available comparing ARBs versus ACE inhibitors with regards to our outcomes of interest is limited. Secondly, some RCTs were of poor quality, for example, were single-center with short duration, and enrolled a few participants. Thirdly, there was significant heterogeneity among the included studies for the outcomes of HOMA-IR, FPI, and DBP, which may result from the differences in trial populations, treatment regimens, and methodological differences. Given the limitations of the included studies, the above conclusions need to be further verified by more high-quality RCTs.

## 5. Conclusion

Taken together, results from this meta-analysis showed that ACE inhibitors resulted in more effective improvement of HOMA-IR compared with ARBs among the long-term intervention subgroup and hypertensive patients with DM subgroup; furthermore, the efficacy of ACE inhibitors on improving GIR and ISI composite was significantly better than that of ARBs in hypertensive patients. However, ARBs had no significant difference in QUICKI score, FPG, FPI, SBP, and DBP compared with ACE inhibitors. Larger and better-designed studies comparing these two RAS system blockers and their effects on IS in the future could hopefully shed better light.

## Supporting information

S1 Checklist PRISMA 2009 checklist. (DOC)
